# Single-cell phenotypes revealed as a key biomarker in bacterial–fungal interactions: a case study of *Staphylococcus* and *Malassezia*

**DOI:** 10.1128/spectrum.00437-23

**Published:** 2023-11-01

**Authors:** Eun Sun Lyou, Min Sung Kim, Soo Bin Kim, MinJi Park, Kyong-Dong Kim, Won Hee Jung, Tae Kwon Lee

**Affiliations:** 1 Department of Environmental & Energy Engineering, Yonsei University, Wonju, South Korea; 2 Bio-Chemical Analysis Group, Centre for Research Equipment, Korea Basic Science Institute, Cheongju, South Korea; 3 Department of Systems Biotechnology, Chung-Ang University, Anseong, South Korea; University of Southern Denmark, Odense, Denmark

**Keywords:** bacterial-fungal interactions, single cell, phenotype, Raman spectroscopy, transcriptomic profile

## Abstract

Microbial interactions are determined by competition, cooperation, and inaction, depending on the transcriptional response to the metabolites secreted by each other. The consequence of these interactions is reflected in the cell phenotype by the composition of cell materials, including lipids, nucleotides, and proteins. We studied the phenotypic plasticity of single cells involved in microbial interactions using Raman spectroscopy and evaluated the types of interactions by linking phenotypes to transcriptional profiles. We used a membrane-based co-culture system to induce chemical interactions between skin-dominant bacteria and fungi, *Staphylococcus* and *Malassezia. Staphylococcus* species exhibited an amensalism interaction with *Malassezia* based on growth and cell viability. *Malassezia* co-cultured with *S. epidermidis* and *S. aureus* resulted in significant changes in the Raman spectra of nucleic acids and proteins in the Raman phenotypes, respectively. The observed differences in phenotypes during co-culture with *Malassezia* were significantly associated with changes in transcriptomic profiles, which demonstrated variations in the defense/resistance and biofilm-formation mechanisms of *S. epidermidis,* whereas no meaningful changes were observed for the transcriptome of *S. aureus*. These approaches provide a robust, simple, and reproducible method for comprehending bacterial–fungal interactions.

**IMPORTANCE:**

Evaluating bacterial–fungal interactions is important for understanding ecological functions in a natural habitat. Many studies have defined bacterial–fungal interactions according to changes in growth rates when co-cultivated. However, the current literature lacks detailed studies on phenotypic changes in single cells associated with transcriptomic profiles to understand the bacterial-fungal interactions. In our study, we measured the single-cell phenotypes of bacteria co-cultivated with fungi using Raman spectroscopy with its transcriptomic profiles and determined the consequence of these interactions in detail. This rapid and reliable phenotyping approach has the potential to provide new insights regarding bacterial–fungal interactions.

## INTRODUCTION

The skin microbiome comprises millions of bacteria, fungi, and viruses (1). As the “second skin,” the skin microbiome plays a crucial role in regulating the immune system and decomposing natural products (1, 2). In addition, the skin microbiome acts as a physical barrier that prevents pathogen invasion (3). In situations where the barrier is broken or the balance between commensals and pathogens is disrupted, skin diseases may occur (3). Understanding microbial interactions in the skin microbiome is essential for maintaining its stability and promoting skin health.

Bacteria and fungi can interact through direct cell–cell contact and chemical communications by secreting small molecules that are involved in defense/resistance, quorum sensing, and biosynthesis mechanisms (4 – 6). These interactions mutually regulate gene expression and cause phenotypic changes. Consequently, they play a key role in balancing the skin microbiome and maintaining skin health (7, 8). Microbial interaction studies primarily involve genotypic and physiological approaches such as transcriptomic analysis and fluorescence *in situ* hybridization (9). However, co-occurrence analysis using typical sequencing-based taxonomic relative abundance provided only an initial evaluation of the major fungal–bacterial relationships (10). Raman spectroscopy with stable isotope probing or nanoscale secondary ion mass spectrometry (nanoSIMS) is currently utilized to access the overall metabolism of microorganisms at the single-cell level and provide phenotypic traits along with biomolecule information (11). Phenotypic traits comprise observable characteristics, such as bacterial cell morphology, reaction, or physiology reflecting the surrounding environment (12). Although phenotypic traits of single cells can be analyzed by several approaches, methods for analyzing them in conjunction with transcriptional profiles are limited. This is because analyzing single cells in mixed populations is often challenging. In membrane-based cultivation, the two species are physically separated. This allows physiological changes caused by chemical interaction to be easily analyzed for phenotype and gene expression (13), and it is a useful and comprehensive approach for observing the phenotypic traits of a single cell in microbial interactions and understanding the relevance of the transcriptional profile.

Fungi rapidly colonize the site of skin wounds when skin balance is disrupted. Kalan et al. (14) demonstrated that fungi are present in 80% of chronic non-healing wounds in patients with diabetes. *Malassezia* species are basidiomycetous fungi, among the most dominant fungal organisms in the skin microbiome (10). The pathogenesis of atopic dermatitis (AD) is strongly associated with *S. aureus*. Their interaction with *M. restricta* is inevitable, as they share the same niche (15). Overgrowth of *S. aureus* in AD lesions appears to be the norm due to a lack of abundance in *Malassezia* (16). In addition, *Malassezia* secretes a protease that rapidly breaks down major virulence proteins of *S. aureus*, thereby inhibiting the formation of *S. aureus* biofilm (17). Although *Malassezia* interactions with bacteria in the skin are closely related to host health, knowledge of phenotypic plasticity is inadequate.

Raman spectroscopy is a single-cell phenotyping technology that measures phenotypic heterogeneity (18) and enables non-destructive, rapid microbial detection and identification through inelastic scattering, and the acquisition of Raman spectra. In contrast to bulk genotypic analysis, which computes average gene expression across all cells in a sample, Raman spectroscopy can accurately capture high-resolution phenotypic heterogeneity through single-cell-level analysis (19). Heyse et al. (13) detected differences in nucleic acids by analyzing the Raman spectra of co-cultures and axenic cultures of *Enterobacter* and *Pseudomonas*.

In this study, we report a new combination of approaches for understanding microbial interactions by linking phenotypic plasticity and transcriptomic profiles in a membrane-based co-culture system. Membrane-based co-culture systems exclude physical interaction and facilitate chemical interaction through metabolic exchange across the membrane. Unlike mixed communities, this co-culture system allows for basic phenotypic studies, such as analyses of growth and viability in isogenic populations. Furthermore, advanced phenotypic studies such as Raman spectroscopy at the single-cell level can be performed. In this study, we used Raman spectroscopy to characterize the variability of the biochemical cellular molecules resulting from the interaction of *Staphylococcus* and *Malassezia*. We investigated the effect of bacterial–fungal interactions on the phenotypes of representative *Staphylococcus* species in the skin microflora. Researchers have hypothesized that different interactions would cause distinct phenotypic changes in each *Staphylococcus* species. To determine whether a difference existed in the interaction, we performed Raman spectroscopy-based phenotype analysis in co-cultures of *M. restricta* with two *Staphylococcus* species. The types of interaction were estimated by analyzing the cell growth and viability of *Staphylococcus* and *M. restricta* and examining the relationship between RNA-Seq and phenotype analysis data. These transcriptomic profiles and phenotype studies provide a new perspective on bacterial–fungal interactions.

## RESULTS

### Positive effects on the growth of *Staphylococcus* in co-culture with *Malassezia* species

**Fig 1 F1:**
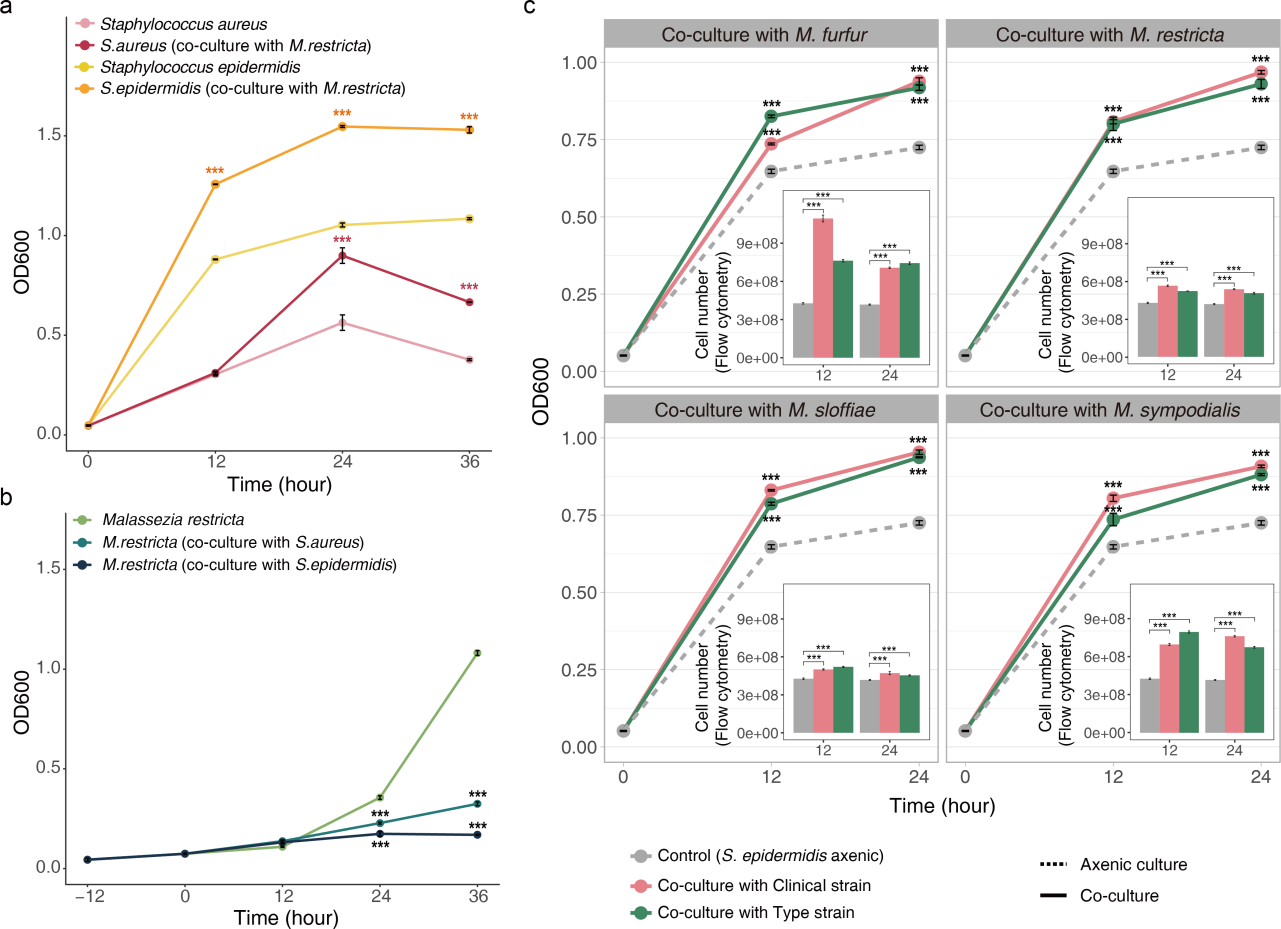
Growth rate (OD600) of co-cultures and axenic cultures. (a) Bacterial growth rate. Co-culture with *Malassezia* promotes the growth of *Staphylococcus*. (b) Fungal growth rate. The presence of *Staphylococcus* species inhibits the growth of *Malassezia*. (c) Co-culturing with different *Malassezia* species equally enhances *Staphylococcus epidermidis* growth. Using flow cytometry, an internal bar graph presents the number of cells per mL. ***P < 0.001 Wilcoxon test (axenic culture vs co-culture).

The growth of *Staphylococcus* strains was observed in the absence or presence of *M. restricta*. The growth rates of the two *Staphylococcus* species under co-culture conditions were higher than those under axenic conditions (Fig. 1a [Fig F1] [Fig F1]). The difference in the growth of *S. aureus* between the axenic cultures and co-cultures was noticeable after 24 h. By contrast, this difference for *S. epidermidis* was noticeable after 12 h. Unlike the *Staphylococcus* species, *M. restricta* showed a lower growth rate under co-culture than that under axenic culture(Fig. 1b). To prove that these trends were not limited to the *M. restricta* strain used in this study, we compared *S. epidermidis* growth rate in co-culture with different species of *Malassezia* (clinical strains: *M. restricta* KCTC 27539, *M. sympodialis* KCTC 27773, *M. furfur* KCTC 27819, and *M. slooffiae* KCTC 27772; type strains: *M. sympodialis* CBS 7222, *M. furfur* CBS 1878, and *M. slooffiae* KCTC 27533). As expected, we observed that co-culture with these seven *Malassezia* species consistently increased the growth rate of *S. epidermidis*, as shown by both optical density and flow cytometric analyses(Fig. 1c). These results indicate that co-culturing with *Malassezia* has a positive effect on the growth of *Staphylococcus*.

### Effect of the presence of *Malassezia restricta* on the viability of *Staphylococcus*

To quantify the impact of co-culture with *M. restricta* on the active cells of *Staphylococcus* species during the exponential phase (12 h) and stationary phase (24 h), we assessed the viability of *Staphylococcus* species using flow cytometry. Differences between live and dead populations of *Staphylococcus* were observed over time in co-cultures and axenic cultures (Fig. S1A). We determined viability by comparing the number of viable cells of *Staphylococcus* strains co-cultured with *M. restricta* to that in the axenic cultures (Fig. S1B). The viability of co-cultured *S. aureus* increased by 0.98-fold and 1.08-fold in 12 and 24 h, respectively, compared to that in the axenic cultures. The live population of co-cultured *S. epidermidis* increased by 1.15-fold and 1.90-fold in 12 and 24 h, respectively. Over time, the difference in viability between axenic and co-cultured cells increased, and this difference was more pronounced for *S. epidermidis* than for *S. aureus*. The variations in *S. aureus* viability under co-culture were comparable to those under axenic culture, indicating that *S. aureus* was less influenced by co-culturing. *M. restricta* for more than 12 h played a favorable role in *Staphylococcus* strain growth and viability, especially in *S. epidermidis*.

Because this study aimed to observe phenotypic changes in bacterial cells caused by fungal–bacterial interactions, the proportion of living cells was critical. Even under axenic conditions, cell viability decreased according to the growth phase. When *S. epidermidis* was cultured for 24 h under axenic conditions, the proportion of live cells was 24.1% (Fig. S1A). For phenotypic and transcriptomic analyses, the culture was stopped at a point when the fraction of live cells was ≥50%. Another factor to consider is whether a sufficient distinction exists between axenic culturing and co-culturing. Considering these factors, the analysis focused on *S. aureus* for 24 h and *S. epidermidis* for 12 hr.

### Effect of *Staphylococcus* species on phenotypic plasticity

**Fig 2:**
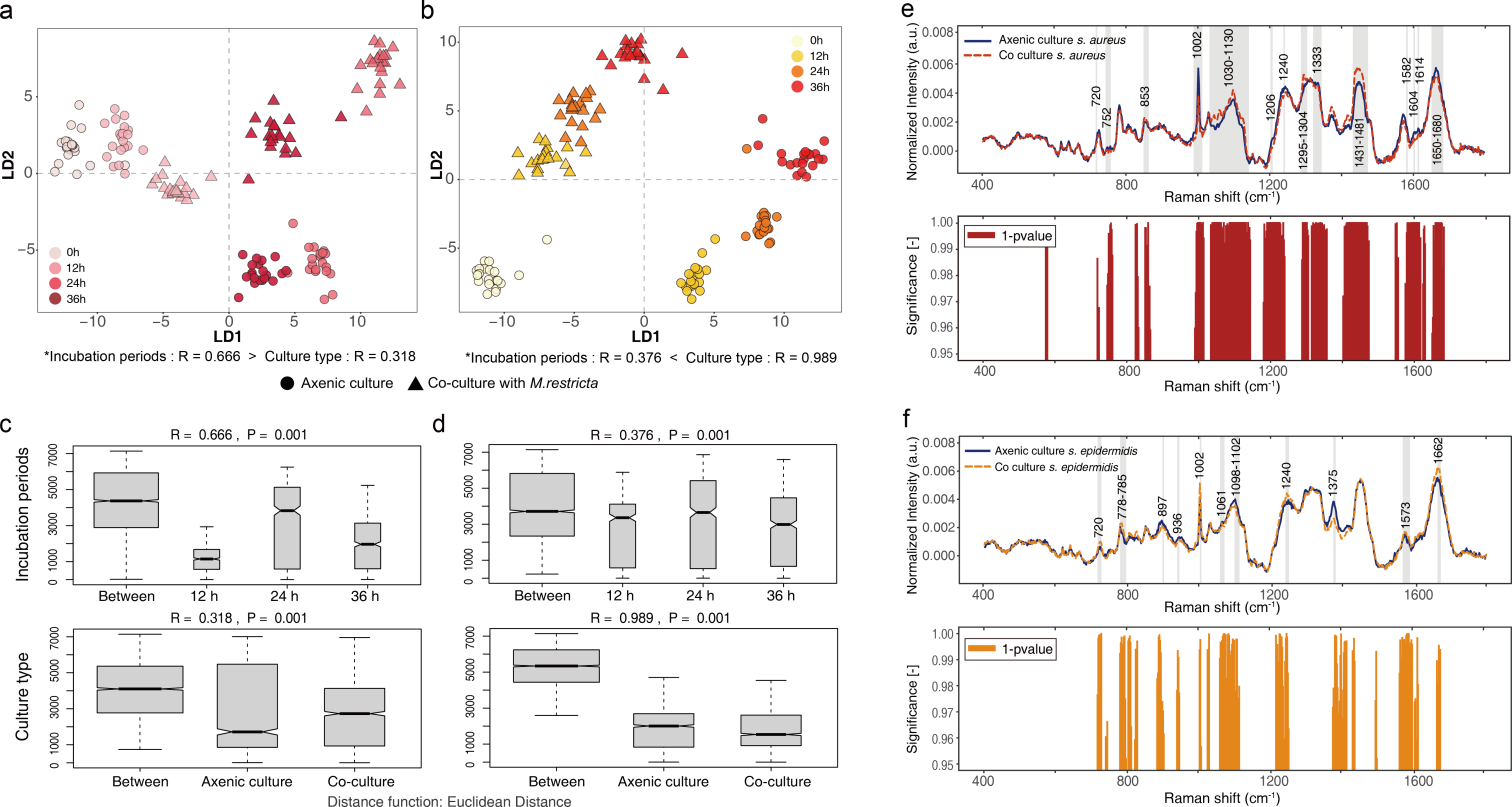
Analysis of bacterial single-cell Raman spectra. Visualization of the separability of the single-cell Raman spectra for (a) *Staphylococcus aureus* and (b) *Staphylococcus epidermidis* in axenic cultures and co-cultures in time series. For each population, 20 single-cell measurements were made. ANOSIM analysis results for (c) *S. aureus* and (d) *S. epidermidis*. Between represents the difference between groups; others are within groups. The greater the distance, the greater the difference. The thickness represents the sample size. Mean spectra of (e) *S. aureus* at 24 h and (f) *S. epidermidis* at 12 h with corresponding significance (1 − *P*-value) of the Student’s *t*-test along the spectral axis. The gray areas indicate the statistically significant Raman shift interval.

We tested the effect of co-culture on phenotypic plasticity using Raman spectroscopy. The Raman spectra of *Staphylococcus* species and *M. restricta* showed significant differences in peaks related to proteins (853 cm^−1^), nucleic acids (782, 1,304, 1,375, and 1,573 cm^−1^), and amides (1,304, 4,573, 1,655, and 1,663 cm^−1^) between the co-cultures and axenic cultures (*t*-test, *P* < 0.05; Fig. S2; Table 1). To determine which culture factors (incubation period and culturing type) affected the phenotype, Raman spectra obtained from each species were individually visualized using discriminant analysis of principal component (DAPC; Fig. 2a and b; Fig. S3A and B). The Raman spectra of the two species of *Staphylococcus* and *M. restricta* (co-cultured with *S. aureus* and *S. epidermidis*) differed for different incubation periods and culture types. The culture factors leading to phenotypic plasticity between clusters were determined by analysis of similarity (ANOSIM; Fig. 2c and d; Fig. S3C and D). With *S. aureus*, the difference in phenotype between incubation periods (ANOSIM, *R* = 0.666, *P* = 0.001) was more significant than that between culture types (ANOSIM, *R* = 0.318, *P* = 0.001). By contrast, with *S. epidermidis,* the difference in phenotype between culture types (ANOSIM, *R* = 0.989, *P* = 0.001) was more significant than that between incubation periods (ANOSIM, *R* = 0.376, *P* = 0.001) (Fig. 2c and d). In other words, the incubation periods dominantly influenced the phenotypic change in *S. aureus*, whereas co-culturing with *M. restricta* influenced the phenotypic change in *S. epidermidis*. The phenotypes of the two *Staphylococcus* species were confirmed to have been proactively changed by different culture conditions. Upon co-culture with *M. restricta*, the Raman peaks associated with proteins (*S. aureus*) and nucleic acids (*S. epidermidis*) composition were significantly altered compared with the peaks obtained from the axenic cultures (*t*-test, *P* < 0.05; Fig. 2e and f; Table 1).

**TABLE 1:** Assignment of the main Raman bands^*a*^

Raman wavenumber (cm ^−1^)	Assignment	Group	Reference	Figure no.
720	Adenine	Adenine	(20)	Fig. 2e and f
752	δ(C–C) Tyr	Protein, cytochrome	(21)	Fig. 2e
778–785	Cytosine, uracil (ring, str)	Cytosine, uracil	(20)	Fig. 2e
853	ν(C–C) proline, ring breath. Tyr	Protein (glycogen, collagen)	(21, 22)	Fig. 2e
897	COC str		(23)	Fig. 2f
936	C–O–C linkage, C–C stretch., α-helix	Carbohydrate, protein	(22)	Fig. 2f
1002	Phenylalanine	Phenylalanine, b-carotene	(24)	Fig. 2e and f
1030–1130	Carbohydrates, mainly –C–C–(skeletal), C–O, def(C–O–H)		(25)	Fig. 2e
1061	C-N and C-C str		(20)	Fig. 2f
1098–1102	Phosphate, CC skeletal, and COC str	Phosphate	(23)	Fig. 2f
1206	Aromatic amino acids	Aromatic amino acids	(26)	Fig. 2e
1240	Thymine, cytosine, adenine, ring ν	Thymine, cytosine, adenine	(27)	Fig. 2e and f
1295–1298	CH2 def, CH2 twist	Saturated lipid	(20)	Fig. 2e
1333	CH3CH2 def. of collagen	Nucleic acid, protein	(28)	Fig. 2e
1375	Thymine, adenine, guanine	Thymine, adenine, guanine	(27)	Fig. 2f
1431–1481	Protein marker band 1451		(27)	Fig. 2e
1573	Guanine, adenine; amide II, C = C, N–H def, and C–N str (amide II)	Guanine, adenine; amide II	(24)	Fig. 2f
1582	Protein	Protein	(20)	Fig. 2e
1604	Phenylalanine	Phenylalanine	(24)	Fig. 2e
1614	Tyrosine	Tyrosine	(20)	Fig. 2e
1650–1680	Amide I	Amide I	(20)	Fig. 2e
1662	Amide I	Amide I	(23)	Fig. 2f

^*a*^ Raman peaks of bacterial spectra and attempt to assign bands based on the literature regarding vibrational energy bands.

The Raman spectra of *M. restricta* were clustered by the same culture factors that contributed to the phenotypic changes in *Staphylococcus* species (Fig. S3). The Raman phenotype of *M. restricta* co-cultured with *S. aureus* showed a greater difference according to the incubation period (ANOSIM, *R* = 0.617, *P* = 0.001) than according to the culture type (ANOSIM, *R* = 0.263, *P* = 0.001). By contrast, the Raman phenotype of *M. restricta* co-cultured with *S. epidermidis* showed a greater difference according to the culture type (ANOSIM, *R* = 0.78, *P* = 0.001) than according to the incubation period (ANOSIM, *R* = 0.264, *P* = 0.001). The alteration in Raman peaks according to the incubation period was greater when co-cultured with *S. epidermidis* than in axenic culture and co-culture with *S. aureus* (Fig. S4). This finding suggests that the phenotypic changes in *Staphylococcus* species can mirror the phenotypic changes in *M. restricta*; however, it is difficult to determine which of the two kingdoms (bacterial or fungal) has a greater influence on the other. The phenotypic changes induced by the chemical interaction between *S. epidermidis* and *M. restricta* are more obvious than those induced by the chemical interaction between *S. aureus* and *M. restricta*.

### Effect of co-culturing *Staphylococcus* with *Malassezia* on the transcriptomic profiles of *Staphylococcus*

**Fig 3:**
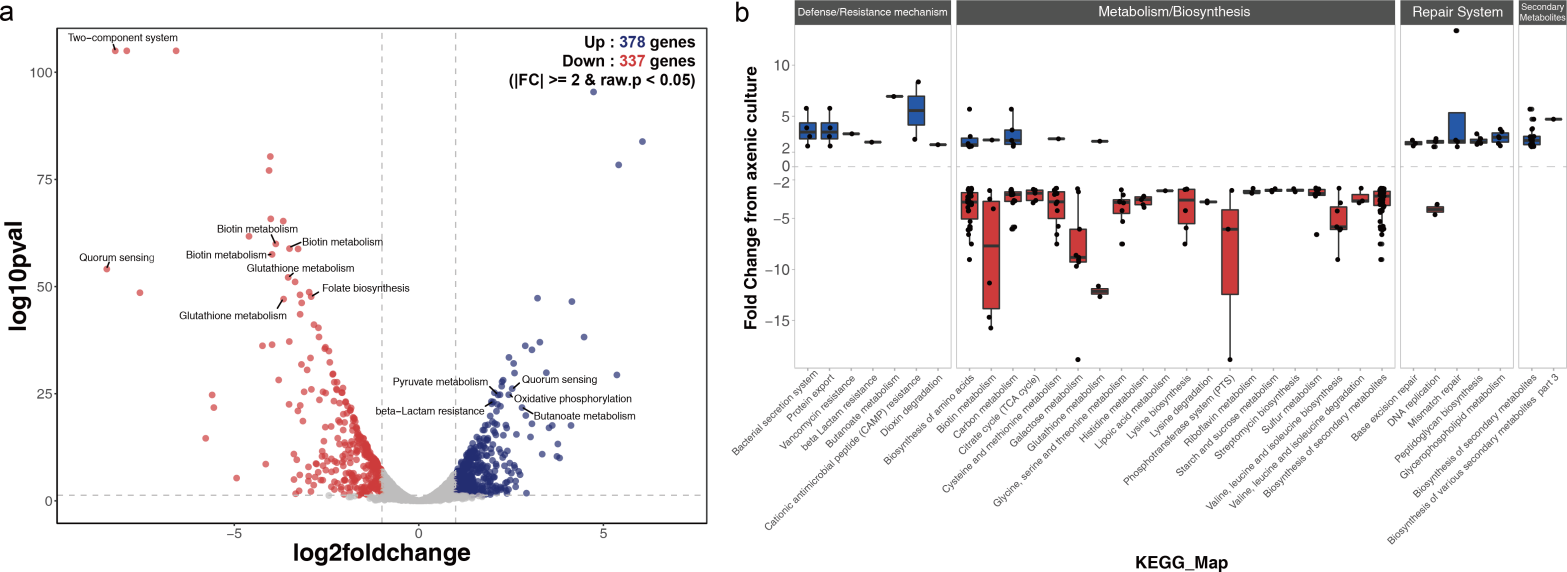
Analysis of various genetic regulatory changes in *S. epidermidis* in the presence of *Malassezia*. (a) Volcano plot shows the magnitude and significance of differentially expressed genes of *Staphylococcus epidermidis* in the axenic culture versus those in the co-culture. Thresholds are shown as dot lines indicating fold changes ≥2 and statistical significance is defined by a *P*-value < 0.05. Functions described in the main text are labeled. (b) The KEGG analysis with significantly enriched pathways in regulated genes from (a) with *P* < 0.05 and ≥2-fold increased expression in *S. epidermidis* (262 genes). Upregulated genes are shown in blue, and downregulated genes are shown in red.

To determine the underlying transcriptomic profiles of phenotypic changes caused by co-culturing, we conducted RNA-Seq analysis of *Staphylococcus* species cultured in the absence or presence of *M. restricta*. A total of 77 and 715 regulated differentially expressed genes (DEGs) were identified in *S. aureus* and *S. epidermidis*, respectively (Fig. 3a; Fig. S5). The results of the RNA-Seq analysis showed that, in the co-culture condition, *S. epidermidis* had approximately nine times more DEGs than *S. aureus*. According to the Kyoto Encyclopedia of Genes and Genomes (KEGG) pathway enrichment analysis, 28 pathways were enriched significantly in *S. epidermidis*. By contrast, only three pathways were observed among DEGs in the transcriptome of *S. aureus* (metabolic pathway, pyrimidine metabolism, and alanine/aspartate/glutamate metabolism) (Fig. S6).

Therefore, we concluded that the influence of *M. restricta* on the transcriptome of *S. aureus* was minimal, supporting the results of the Raman analysis that co-culturing did not significantly affect the phenotype of *S. aureus*. At the transcript level, we confirmed the changes observed in the Raman analysis for the intracellular components of *S. epidermidis* with *M. restricta* co-culture (phenylalanines, adenines, phosphates, amides, and nucleic acids) (Fig. S7). The enriched KEGG pathways with significant DEGs in *S. epidermidis* were classified into four main categories: metabolic/biosynthesis, defense/resistance mechanisms, repair systems, and secondary metabolites (Fig. 3b). The upregulated DEGs were highly associated with three categories: defense/resistance mechanisms (14 genes), repair systems (23 genes), and secondary metabolites (18 genes), indicating that they were overexpressed when exposed to a new environment, such as abiotic stress and competitor appearance. The downregulated DEGs were enriched in metabolic/biosynthesis genes (191 genes), indicating that the pattern of cell physiology, such as biofilm reduction, changed rapidly.

**Fig 4:**
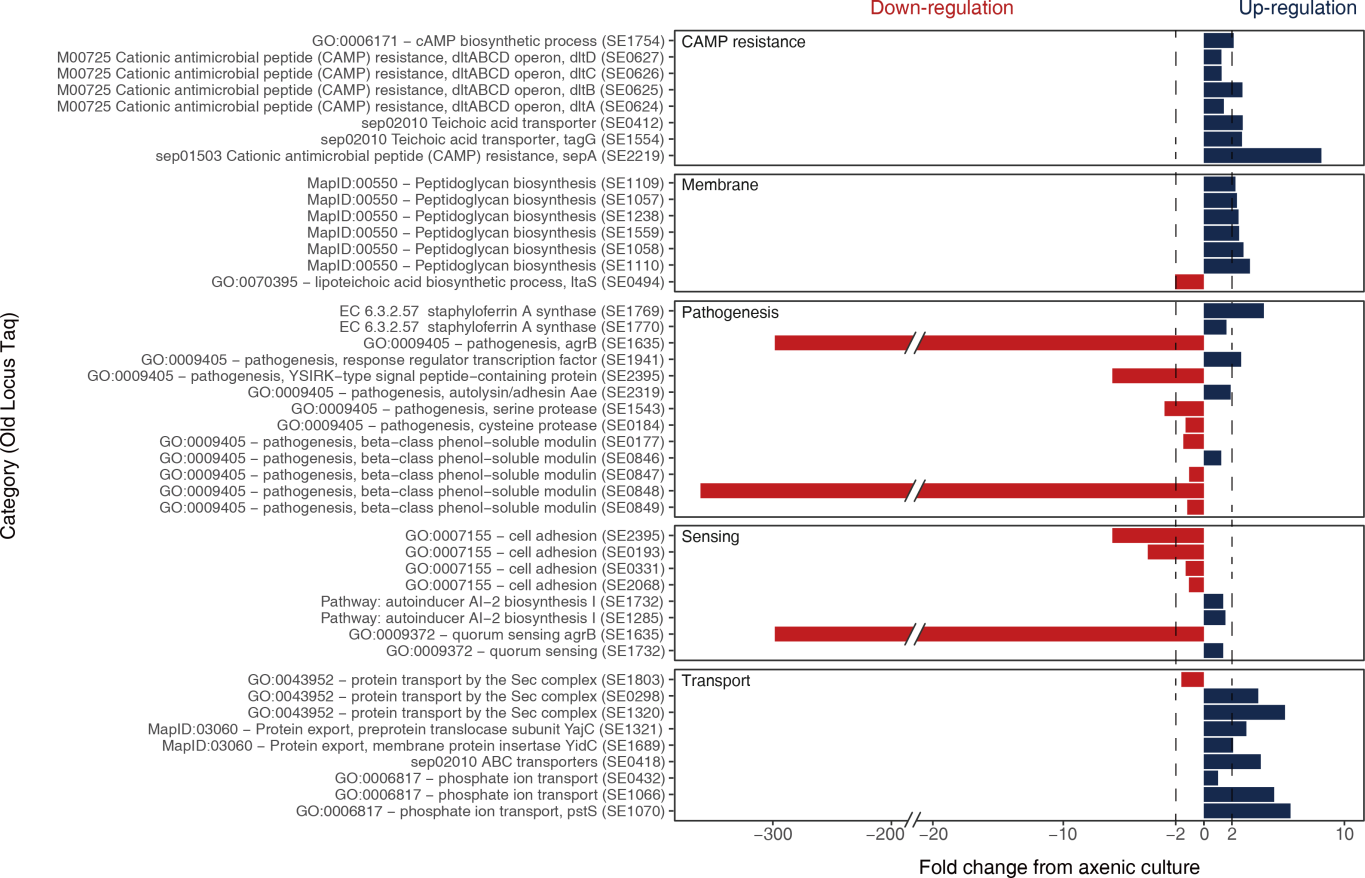
Regulated gene sorting. The KEGG (module, pathway, and enzyme), Gene Ontology term, and Pathway were used to avoid missing differentially expressed genes related to the function. Thresholds are shown as dotted lines, indicating fold changes ≥ 2. Upregulated genes are shown in blue, and downregulated genes are in red.

**Fig 5:**
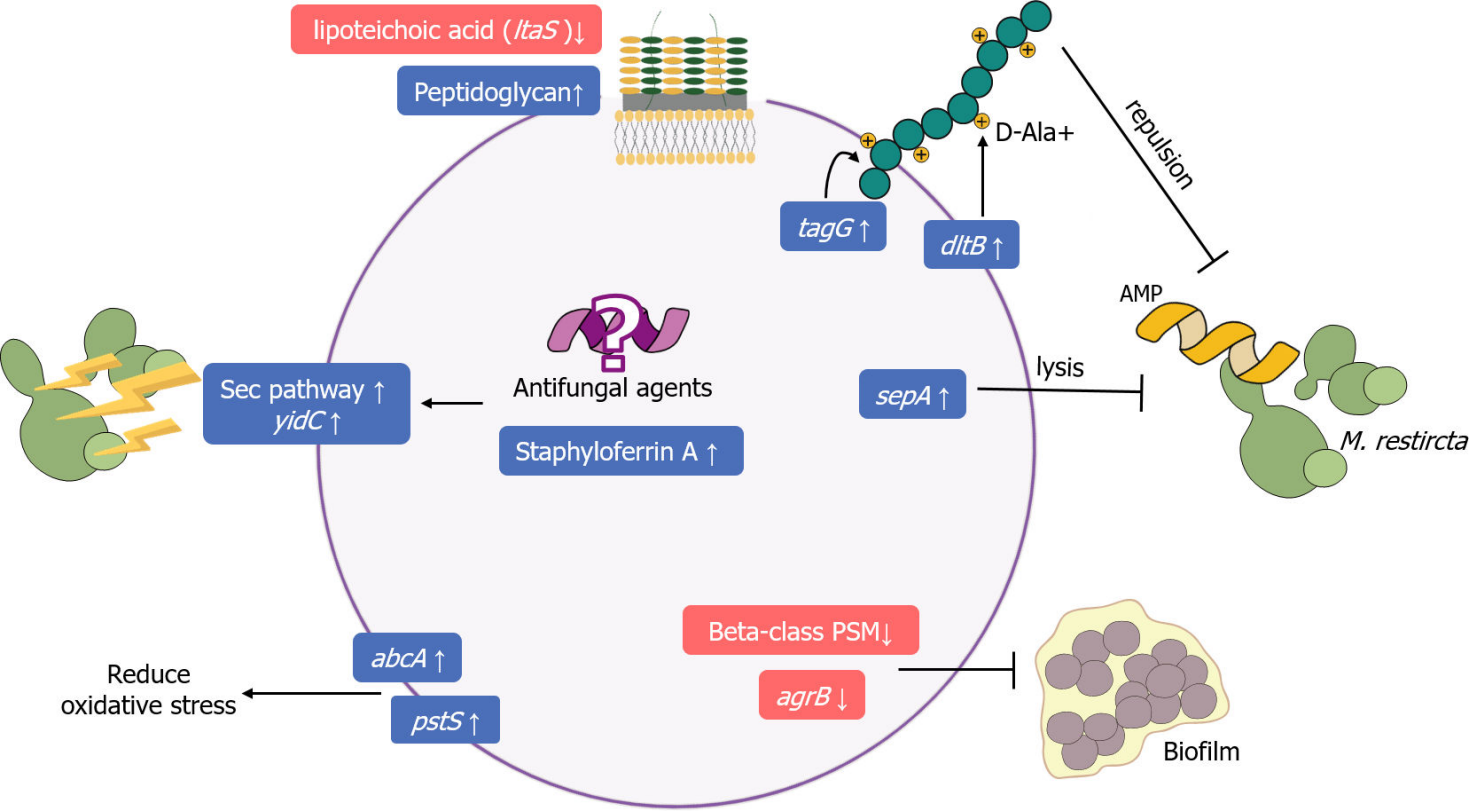
Conceptual model of *Staphylococcus epidermidis* harboring physiological features (i.e., genes and pathways) associated with *Malassezia restricta* co-cultures. Upregulated gene levels are shown in blue boxes, and downregulated gene levels are in red boxes.

Among the DEGs that were upregulated in *S. epidermidis*, we focused on those enriched in cationic antimicrobial peptide (CAMP) resistance, membrane, and transport because these could serve as important factors in microbial interactions (Fig. 4). The expression of *tagG* and *dlt* operons was upregulated by 1.3- to 2.7-folds. In addition, the expression of the gene regulating CAMP lysis, *sepA*, showed 8.4-fold upregulation. Furthermore, upregulation was observed for the following genes associated with transport/secretion mechanisms: the Sec/YidC pathway genes (SE1320, SE0298, and *yidC*; 2.1- to 5.8-fold upregulation), a phosphate ion transport gene (*pstS*; 6.2-fold upregulation), and an ABC transporter gene (*abcA*; 4.0-fold upregulation). The peptidoglycan synthesis-related genes showed 2.2- to 3.3-fold upregulation. Unlike the upregulated expression of peptidoglycan metabolism genes, the expression of the *ltaS* gene was downregulated by 2.0-fold. The expression of the secretion-related transporters and staphyloferrin A, a type of siderophore, was simultaneously upregulated (SE1769 and SE1770; 1.6- to 4.7-fold upregulation). One of the most downregulated genes was *agrB* (298.6-fold downregulation). The expression of the beta-class phenol-soluble modulin (PSM) gene (SE0848) showed 350.0-fold downregulation. Since these two genes are key regulators of biofilm formation and development, a drastic decrease in their expression leads to a decrease in biofilm. The crystal violet assay confirmed a considerable reduction in the biofilm produced from *S. epidermidis* in the co-culture with *M. restircta* compared with that in the axenic culture (Fig. S8). To investigate the bacterial–fungal interactions more intuitively, we propose that *S. epidermidis* interacts with *M. restricta*, based on their transcriptional profiles (Fig. 5). Co-culturing with *M. restricta* allows *S. epidermidis* to change its physiology to increase its fitness through actions such as preventing chemical attacks on coexisting species and reducing biofilm formation.

## DISCUSSION

*S. aureus* and *S. epidermidis* exhibited an increase in growth and viability when co-cultured with *M. restricta*. Such physiological effects observed in membrane-based co-culturing systems indicate chemical interactions in which one microbe produces a useful or harmful metabolite for the other microbe (29). *S. aureus* was primarily associated with changes in peaks related to proteins and carbohydrates, which are strongly associated with biofilm formation (30). By contrast, *S. epidermidis* was associated with peaks related to nucleic acids, indicating bacterial cell proliferation or gene expression (31). The changes in nucleic acid composition in *S. epidermidis* suggest that the co-culture with *M. restricta* actively changed the transcription profile of *S. epidermidis*. These results suggest that phenotyping *via* Raman spectroscopy enabled the identification of physiological response patterns in bacterial–fungal interactions in two *Staphylococcus* species by offering single-cell resolution phenotypic information that cannot be provided by basic phenotypic parameters, such as growth (32).

We compared the phenotypes based on co-culture conditions and incubation periods, using Raman spectroscopy to understand the effect of bacterial–fungal interactions on the *Staphylococcus* species. The intensity of Raman peaks of the lipids, carbohydrates, proteins, and nucleic acids that were common to both species changed according to the incubation periods, as shown by the time-to-time ANOVA analysis. This finding is consistent with a study by Kochan et al. (33), who observed similar changes in Raman peaks according to the growth phase. These results are consistent with the findings of a previous study, which used DAPC to visualize the cluster separations of Raman spectra in the axenic cultures according to incubation periods. Interactions between the bacteria and fungi were categorized as passive and active. Passive interactions refer to the phenotypic changes that were more significantly influenced by the incubation period than by the co-culture conditions. Active interactions were defined as a situation in which the phenotypic change induced by the co-culture conditions surpassed that induced by the incubation period. When co-culturing with *M. restricta*, the *R*-value of the incubation period was approximately half that of *S. aureus*, indicating a passive interaction. In contrast, *S. epidermidis* had an *R*-value approximately three times that of the incubation period, indicating active interaction. *M. restricta* demonstrated the same interaction as that observed in co-cultured *Staphylococcus*. In this study, the *S. aureus* and *S. epidermidis* strains used were coagulase-positive and coagulase-negative *Staphylococci*, respectively. Because differences exist in defense mechanisms (34, 35), we expect variations in the response of other microorganisms to metabolites.

In conclusion, our investigation of the phenotypic traits of *Staphylococcus* that chemically interact with fungi using a membrane-based culture system has helped us understand the active participation of *Staphylococcus* in the interaction with *Malassezia*. This understanding can be further improved by analyzing the type and extent of changes in cellular components.

We conducted RNA-Seq to identify a reliable link between gene expression and phenotype under co-culture conditions to explain the effect of co-culture on phenotypic changes in *Staphylococcus*. Significant changes were observed in the DEGs of *S. epidermidis* for defense/resistance mechanisms, metabolism/biosynthesis, repair systems, and secondary metabolism in the presence of *M. restricta*, unlike in *S. aureus*, where gene expression remained largely unchanged. The magnitude of these changes in gene expression was highly correlated with the increase in nucleic acid levels, as per the Raman phenotype, in *S. epidermidis* co-cultured with *M. restricta*. The differential expression of many defense-responsive genes during bacterial–fungal interactions may be attributed to the activation of defense signaling against antibacterial agents originating from fungal species (36). Fungi produce CAMPs such as plectasin and copsin, efficiently killing bacteria with negatively charged membranes (37, 38). The notable increase in the expression of CAMP resistance-related genes modifies membrane properties (e.g., charge and structure) through D-alanine and teichoic acid, which enhance the resistance of *S. epidermidis* to CAMP. This may indicate that it responds to the antibacterial effects of *M. restricta* (39). In addition, *S. epidermidis* actively interacts with *M. restricta* by upregulating the synthesis of aureolysin (*sepA*), a zinc metalloproteinase that lyses AMP, and transporters (*pstS* and *abcA*) that reduces oxidative stress (40, 41). We confirmed that multiple defensive mechanisms are involved in fungal resistance during antagonistic cross-talk between *S. epidermidis* and *M. restricta*. However, this study found no direct evidence regarding the genetic expression of antifungal agents. The findings revealed high expression of the YidC/Sec pathway components, which facilitate the secretion of small virulence proteins. However, proteins that act as antifungal agents remain unclear (41). Staphyloferrin A, which is greatly upregulated as a siderophore, plays an important role in indirect competition, affecting the antagonistic effects of *M. restricta* (42). These antagonistic mechanisms might contribute to the observed amensalism and should be considered in future studies. Together, these findings suggest that *S. epidermidis*, a constituent of the normal skin flora, may play a more crucial role in controlling the abundance or activity of *M. restricta* compared with the opportunistic pathogen *S. aureus* in the skin.

Co-culturing with *M. restricta* significantly downregulated the *agrB* gene expression in *S. epidermidis*. The accessory gene regulator (agr) system is a peptide quorum-sensing system that uses peptides and is present in all *Staphylococcus* species, and it is a dominant regulator of biofilm development (43). PSMs act as the main molecular effectors of staphylococcal biofilm maturation. Their hypothesized functions include promoting skin colonization, nutrient emulsification, antibiotic effects, and cytotoxicity. However, in this study, they were highly repressed in the transcriptomic profiles (44, 45). The downregulation of the agr system and PSMs suggests that *S. epidermidis* preferred to form planktonic cells rather than biofilm due to its antagonistic interaction with *M. restricta*. Moreover, the standard crystal violet assay for biofilm biomass demonstrated that *M. restricta* inhibited co-cultured *S. epidermidis* from producing biofilm (Fig. S8). Such an explanation accounts for the insignificant increase in protein and carbohydrate levels in the Raman phenotype of *S. epidermidis*, unlike that of *S. aureus*. These findings suggest that Raman spectroscopy can indirectly measure the phenotypic traits of single cells of *S. aureus* attempting to engage in biofilm formation based on the Raman spectral intensity of proteins and lipids. Changes in cell physiology induce structural modifications in the cell membrane and upregulation of peptidoglycan (PG)-related genes, which determine the bacterial shape and strength (46). The increase in the amide peak (47) reflects this phenomenon. In addition, cell physiology changes caused a decrease in the expression of lipoteichoic acid (LTA). In gram-positive bacteria, PG is a key determinant of the structural characteristics of the membrane depending on their growth state, either biofilm or planktonic growth state (48). LTA and PG are roughly in equal amounts in gram-positive bacteria, and LTA plays an important functional role in biofilm formation (49). The change in the ratio of PG and LTA in the cross-linking process represents a survival strategy that responds to antibacterial substances in a growth state where *Staphylococcus* does not form a biofilm (50). The modified structure of the cell membrane without biofilm formation in the presence of *M. restricta* may enhance the overall resistance of *S. aureus* to fungal antibacterial effects during the interaction, as suggested by the distinct physiology of the bacteria.

This study explored bacterial–fungal interactions under batch conditions, providing a novel perspective on understanding the skin microbiome, despite the differences from interactions in the real skin environment. While *S. epidermidis* and *M. restricta*, the normal skin flora, exhibit significant phenotypic and transcriptional interactions, *S. aureus*, an opportunistic pathogen, displays independent physiological characteristics that are unaffected by the presence of *M. restricta*. These bacterial–fungal interactions may significantly enhance our understanding of skin health, disease progression, and treatment response. The insights obtained here could help guide the development of more effective and targeted interventions, including probiotics, for skin disorders. This study highlights the potential of using these interactions as phenotypic or transcriptional markers for developing skin therapeutics that selectively target pathogens while minimizing their impact on normal skin flora. Considering the phenotypic or transcriptional characteristics of normal skin flora, rather than just reducing the presence or abundance of pathogens, treatments can aid patients in achieving a healthy skin microbiome.

In summary, this study presents the first proof-of-concept for the successful use of single-cell phenotyping to understand the cell physiology of microbial interactions. Transcriptomic profiles were used to comprehensively interpret the phenotypic information with high sensitivity. Two species of *Staphylococcus* interacted amensally with *M. restricta*; however, the cellular physiological changes involved in their interaction significantly differed for each species. According to the Raman phenotypes and transcriptomic profiles, compared with *S. aureus*, *S. epidermidis* was confirmed to exhibit considerably more dependent and antagonistic interactions with *M. restricta*. We discovered that *S. epidermidis* reacted aggressively to *M. restricta* by regulating the expression of genes related to defense/resistance mechanisms and biofilm formation. These impressive results demonstrate that Raman spectroscopy has revolutionized the guidance of microbial interaction through fast and reliable phenotypic results at the single-cell level. Further research with various microbial species and different interactions is necessary to enhance understanding and assess the stability of a microbiome, which can be coupled with orthologous omic approaches.

## MATERIALS AND METHODS

### Strains and culture conditions

We used the bacterial strains *S. aureus* NCTC 8325–4 and *S. epidermidis* ATCC 12228, and the fungal strain *M. restricta* KCTC 27527. The effect of *Malassezia* species on the growth rate of *S. epidermidis* was confirmed by co-culturing *S. epidermidis* with seven different species of *Malassezia*. Clinical and type strains were evaluated separately to determine differences in pathogenicity. The following strains were used: *M. restricta* KCTC 27539, *M. sympodialis* KCTC 27773, *M. furfur* KCTC 27819, *M. slooffiae* KCTC 27772, *M. sympodialis* CBS 7222, *M. furfur* CBS 1878, and *M. slooffiae* KCTC 27533. All strains used in this study were obtained from the Korean Collection for Type Cultures and were revived. The strains were axenically cultured or co-cultured in a modified Dixon (mDixon) medium at 34°C and 45 rpm for 36 h. The medium consisted of (g/L): 36 g of malt extract, 6 g of mycological peptone, 20 g of desiccated ox bile, 10 mL of Tween 40, 4 mL of glycerol 50%, and 2 mL of oleic acid. The medium was sterilized by autoclaving (51). The final pH value was adjusted to 6.0.

### Experimental setup

**Fig 6:**
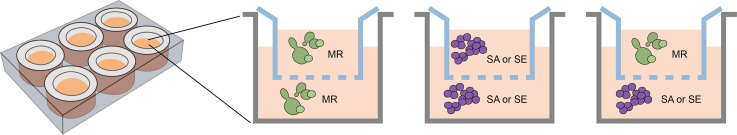
Illustration of the experimental setup. Bacteria and fungi interact *via* metabolites in their shared medium. At the same time, the membrane of the cell culture insert physically separates them. Five synthetic conditions were applied: three axenic cultures and two bacteria–fungi co-cultures. Six biological replicates were used for each synthetic condition. *Malassezia restricta* (MR), *Staphylococcus aureus* (SA), and *Staphylococcus epidermidis* (SE).

We used 24 mm Transwell inserts and plates with 0.4 µm pores (Corning Inc., Arizona, USA) to establish the membrane-based co-culturing system. Five synthetic conditions were created: three axenic cultures and two bacterial–fungal co-cultures (Fig. 6). Each condition was replicated six times. *M. restricta* pre-cultures were diluted to an OD_600_ of 0.05 in medium and grown for 12 h at 34°C. The *Staphylococcus* strains were diluted to an OD_600_ of 0.05 in mDixon. Each 1.5 mL of the identical species suspension was loaded into the insert and plate of each well in a Transwell plate for axenic culturing. Co-culturing was performed by loading 1.5 mL of *M. restricta* onto the inserts and 1.5 mL of *Staphylococcus* species onto the plates. The initial cell densities were set to an OD_600_ of 0.05 in all cultures. No additional medium was added after setting up the experiment. All cultures were incubated for 36 h at 34°C with gentle shaking to facilitate the diffusion of metabolites between compartments. The culture samples were collected every 12 h for downstream analyses, including the measurement of OD_600_, Raman spectroscopy, flow cytometry, RNA-Seq, and biofilm-formation assay.

### Raman spectroscopy

The bacterial cells were washed in phosphate-buffered saline (PBS) by centrifugation at 16,000× *g* for 5 min. The samples were fixed with 4% formaldehyde at 4°C for 2 h and washed twice with PBS buffer. A sample of 1.5 µL of the fixed bacterial cells was spotted on an aluminum-coated slide (LiMedIon, Mannheim, Germany) and air-dried at 25°C. The slide was washed with ultrapure water to remove salts and air-dried. Raman spectra were acquired using the XperRam35V confocal Raman imaging system (Nanobase, Seoul, South Korea) equipped with a 1,800 g/mm grating, a 532 nm neodymium–yttrium aluminum garnet laser, the LTGL-532RL (Leading Tech, Shanghai, China), and an MPLFLN 40× objective (Olympus, Tokyo, Japan). The laser power of a single cell was 2.0 mW. The spectra were integrated for 25 s for each cell. The resulting scattered light was captured on an Atik 428EX color CCD Camera (Atik Cameras, Bawburgh, UK) cooled at −70°C.

### Flow cytometry

To stain the bacterial cells, we used the LIVE/DEAD BacLight bacterial viability and counting kit (Invitrogen, OR, USA) containing SYTO 9 and propidium iodide (PI) for flow cytometry. The samples were diluted 1:100 in PBS and stained with 0.15 vol% (3.34 mM) SYTO 9 for total cell analysis and with 0.15 vol% vol/vol of 30 mM PI for live/dead analysis. The staining was performed following the manufacturer’s instructions. The samples were analyzed using a CytoFLEX V0-B3-R2 flow cytometer (Beckman Coulter, CA, USA) equipped with two scatter detectors, five fluorescence detectors, and two lasers (blue 488 nm laser and red 638 nm laser). Fluorescence was collected in the green (FITC-A/FL1) and red (PE-A/FL2) channels, which are filters for detecting viable cells. Populations of live and dead bacteria were gated according to a single SYTO9 and PI stain. A minimum of 30,000 cells were analyzed per sample.

### Biofilm-formation assay

Biofilm formation was assessed in 24 mm Transwell plates with 0.4 µm pores (Corning Inc., Arizona, USA). All conditions were performed under the same conditions as described under “Experimental setup” above. The medium was then discarded, and the wells of each plate were gently washed three times with 2 mL of distilled water (filtered by a 0.25 µm pore size filter), dried at room temperature for 20 min, and subsequently stained with 2 mL of 1% crystal violet for 10 min. Each well was washed three times with 2 mL of distilled water, then dried at 37°C for 30 min. Next, 2 mL of 98% ethanol was added and eluted in an orbital shaker for 2 h. Transferred to a 96-well microplate, and the absorbance at 570 nm was measured using a microplate reader. We used wells filled with mDixon as blanks to correct for background staining by subtracting the blank value from each experimental value.

### RNA extraction and sequencing

We performed RNA-Seq to characterize the transcriptome profile of the *Staphylococcus* bacteria in co-cultures with *M. restricta* and in axenic cultures. The total RNA was extracted using the Maxwell 16 LEV simplyRNA Tissue Kit (Promega, WI, USA) with TRIzol reagent (Invitrogen, MA, USA) according to the manufacturer’s instructions. The total RNA integrity was assessed using the Agilent Technologies 2100 Bioanalyzer (Agilent, CA, USA). RNA-Seq was performed at MacroGen Inc. (Seoul, Korea) using the NovaSeq 6000 platform (Illumina, CA, USA). Libraries were constructed using the TruSeq Stranded Total RNA (NEB Microbe) Kit (Illumina). The prepared libraries were quantified using the Illumina qPCR Quantification Protocol Guide.

### Raman spectral processing

Raman spectral pre-processing was performed using the R package “ChemoSpec” (v5.3.11) (https://bryanhanson.github.io/ChemoSpec/) for baseline correction (function: als), normalization (function: TotInt), and alignment. Raman spectrum analysis was based on DAPC in the R package “adegenet” (52). In DAPC, the data were initially transformed using principal component analysis, and clusters were subsequently identified using discriminant analysis. The region selected for the analysis was 400–1,800 cm^−1^.

### RNA-Seq data alignment and transcriptome profile determination

The reads were aligned to each reference genome (*S. aureus* reference; GCF_000013425.1_ASM1342v1, *S. epidermidis* reference; GCF_000007645.1_ASM764v1) using the Bowtie 1.1.2 alignment software (53). Raw read counts were generated using HTseq (version 0.10.0) (54) for each annotated gene. DEG analysis was performed using EdgeR (version 3.8.6) for comparative combinations (axenically cultured and co-cultured samples). Genes with a false discovery rate (FDR)-adjusted *P*-value < 0.05 and a twofold change exhibited significant differences in expression in the pairwise comparison between the axenic- and co-culturing samples. The Gene Ontology (http://geneontology.org/) and KEGG pathways (https://www.genome.jp/kegg/) were used as functional annotation databases. A DEG list was created by gene set enrichment analysis using several functional Databases for Visualization and Integrated Discovery (https://david.ncifcrf.gov/) tools and the KEGG database.

### Statistics and reproducibility

Statistical analyses were performed using R v4.0.5 (R Foundation for Statistical Computing, Vienna, Austria). The robustness of the DAPC grouping patterns was assessed by the ANOSIM using the Euclidean distance. *t*-Tests were performed for each Raman shift to compare the mean spectral intensities between the axenic cultures and co-cultures of *Staphylococcus*. Raman shifts over the incubation periods of each species were analyzed using an analog of the standard univariate ANOVA for comparing the mean spectral intensities. All *P*-values and FDR-adjusted *P*-values (Benjamini and Hochberg) < 0.05 were considered statistically significant. All data were obtained from at least three experiments, and error bars were produced using the standard error of the mean.

## Data Availability

Denoised raw flow cytometry data can be accessed via FlowRepository (ID: FR-FCM-Z568). The data sets presented in this study can be found online in the NCBI Sequence Read Archive under the Bioproject ID PRJNA821337 for *Staphylococcus aureus* and *Staphylococcus epidermidis*. The data sets generated and/or analyzed during the current study are available from the corresponding author upon reasonable request.

## SUPPLEMENTAL MATERIAL

Fig S1-S8, Table S1 spectrum.00437-23-s0001.pdf Supplemental figures and table.

ASM does not own the copyrights to Supplemental Material that may be linked to, or accessed through, an article. The authors have granted ASM a non-exclusive, world-wide license to publish the Supplemental Material files. Please contact the corresponding author directly for reuse.
