# Supplementary material for: Single-cell phenotypes revealed as a key biomarker in bacterial–fungal interactions: a case study of Staphylococcus and Malassezia
Source: Microbiol Spectr. 2023 Nov 1;11(6):e00437-23. doi: 10.1128/spectrum.00437-23 (PMC10714763; doi:10.1128/spectrum.00437-23)
Supplement: Fig S1-S8, Table S1 — Supplemental figures and table. [file spectrum.00437-23-s0001.pdf]

Supplemental Information

Single-cell phenotypes revealed as a key biomarker in bacterial–fungal interactions: A case study of Staphylococcus and Malassezia

Eun Sun Lyou[1], Min Sung Kim[1,3], Soo Bin Kim[1], MinJi Park[2], Kyoung-Dong Kim[2], Won Hee Jung[2] and Tae Kwon Lee[1, *]

[1]Department of Environmental & Energy Engineering, Yonsei University, Wonju, 26493, Republic of Korea

[2]Department of Systems Biotechnology, Chung-Ang University, Anseong, Korea.

[3]Bio-Chemical Analysis Group, Center for Research Equipment, Korea Basic Science Institute, Cheongju 28119, South Korea

* Corresponding author:

E-mail addresses: tklee@yonsei.ac.kr (T. K. Lee)

Phone: +82-33-760-2446, Fax: +822-33-760-5286

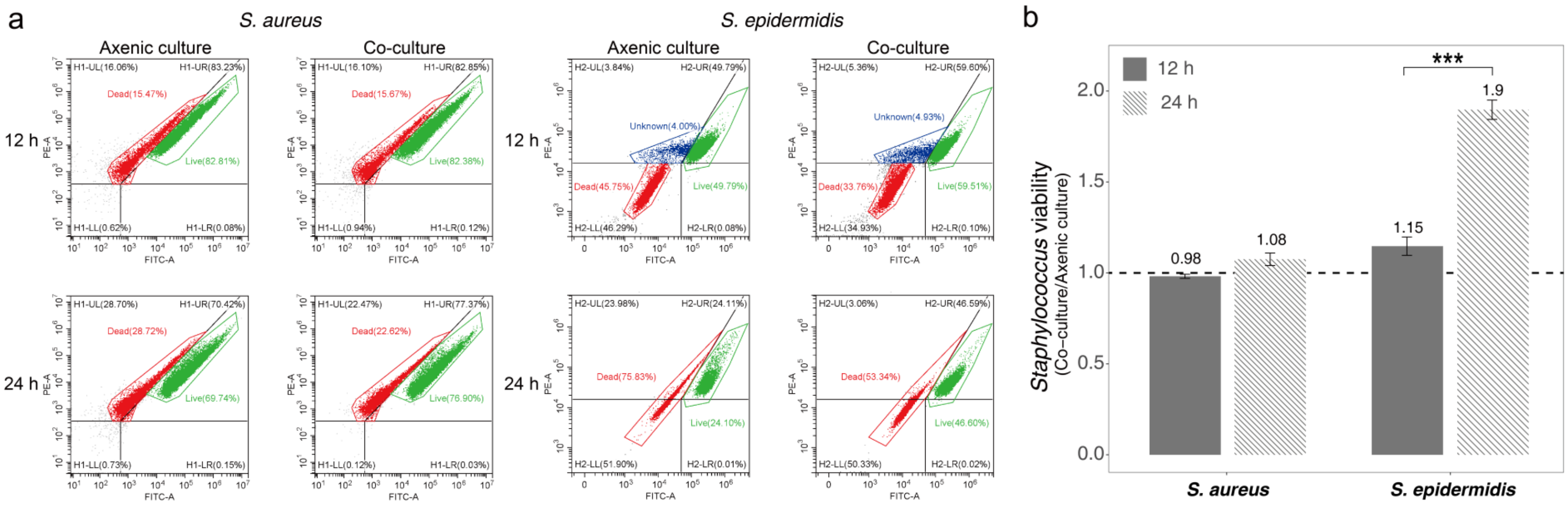

Fig. S1. a Signals for SYTO9 (X axes) and PI (Y axes) are plotted to show the sorting of *Staphylococcus aureus* and *Staphylococcus epidermidis* when cultured axenic and co-cultured for 12 hours, axenic and co-cultured for 24 hours. **b** The viability was estimated by dividing the bacterial live percent in co-culturing by bacterial live percent in axenic culturing for each strain using (**a**) SYTO9-PI results. The error bars represent standard deviation of *Staphylococcus* viability. Co-culturing with *Malassezia restricta* improved the viability of *Staphylococcus* species and increased the viability of *Staphylococcus* species compared to axenic culturing over time. ***$P < 0.001$ Wilcoxon test (Axenic vs. Co-culture).

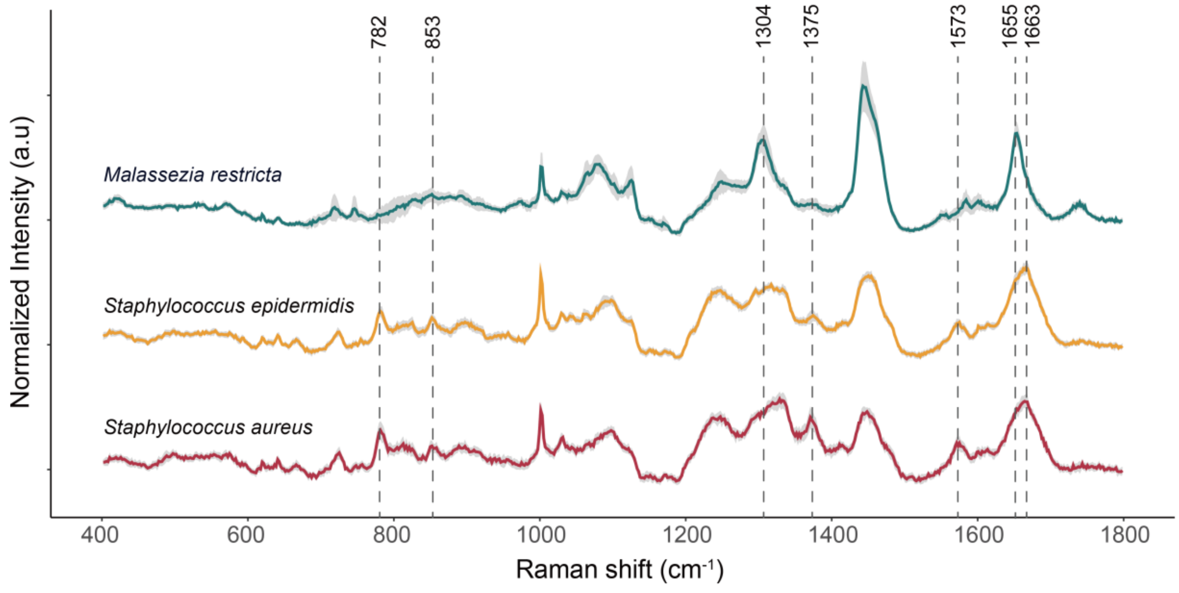

Fig. S2. Average Raman spectra of one *M. restricta* and two *Staphylococcus* species. The gray areas manifest the standard deviations.

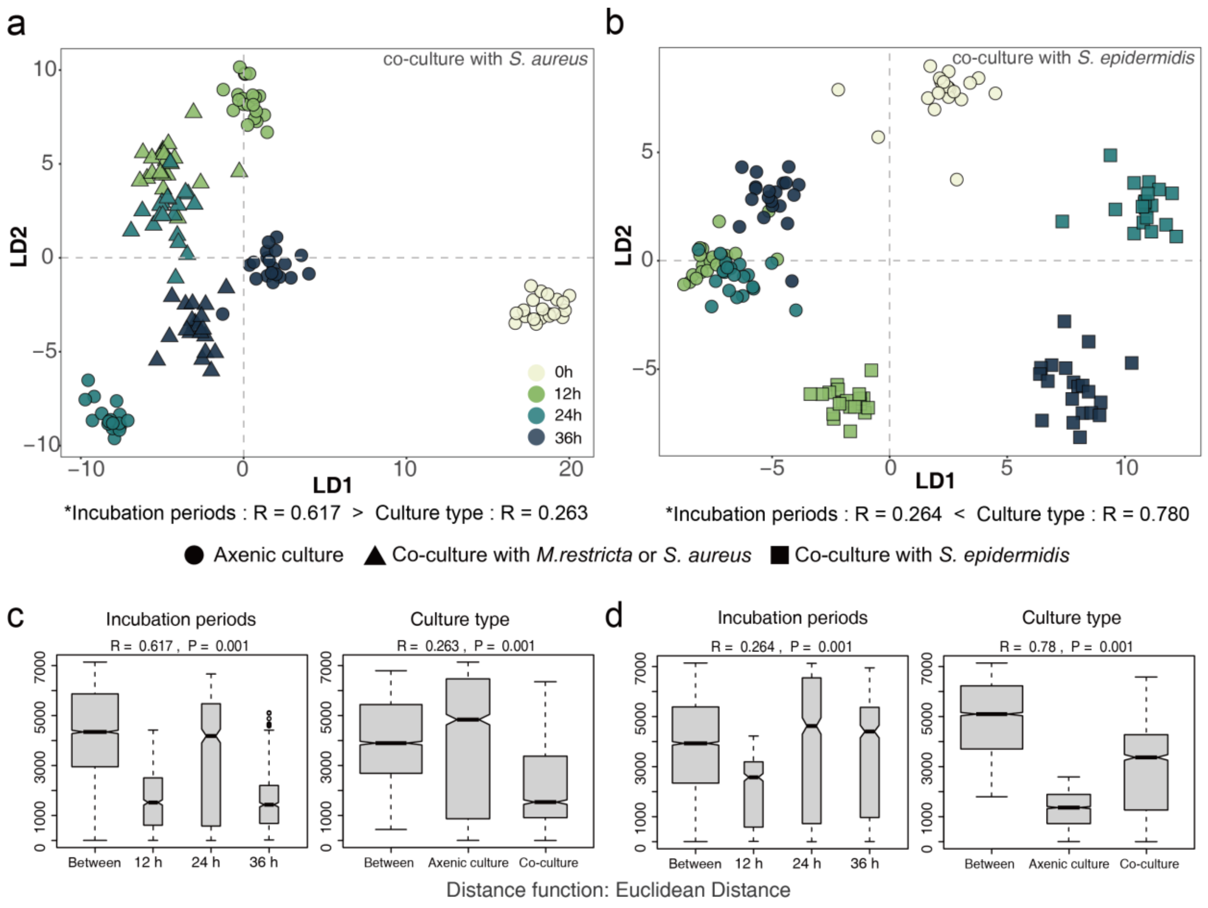

Fig. S3. Visualization of the separability of the single-cell Raman spectra for (**a, b**) *Malassezia restricta* in axenic culturing and co-culturing in time series. There are 20 single-cell measurements for each population. Anosim analysis results of *Malassezia restricta* co-culture with (**c**) *Staphylococcus aureus*, (**d**) *Staphylococcus epidermidis*. Between represents the difference between groups; others are within groups; the greater the distance is, the greater the difference is; and the thickness is the sample size. Between represents the difference between groups; others are within groups; the greater the distance is, the greater the difference is; and the thickness is the sample size.

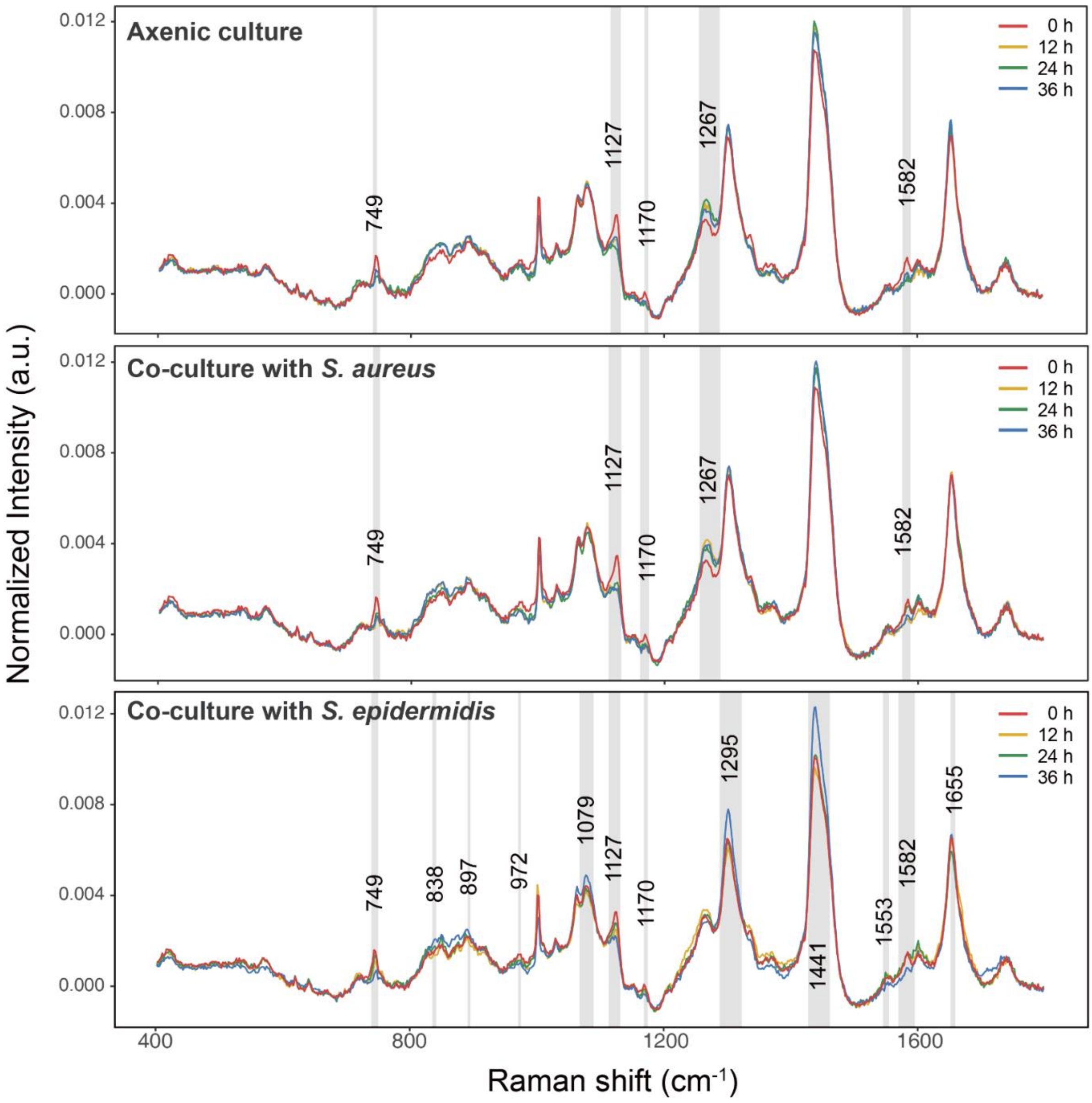

Fig. S4. ANOVA analysis identified peaks in *M. restricta* that vary significantly with incubation periods. A significant changes in axenic culturing were similar in co-culturing with *S. aureus* and different from *S. epidermidis*. The gray areas manifest the statistically significant Raman shift interval.

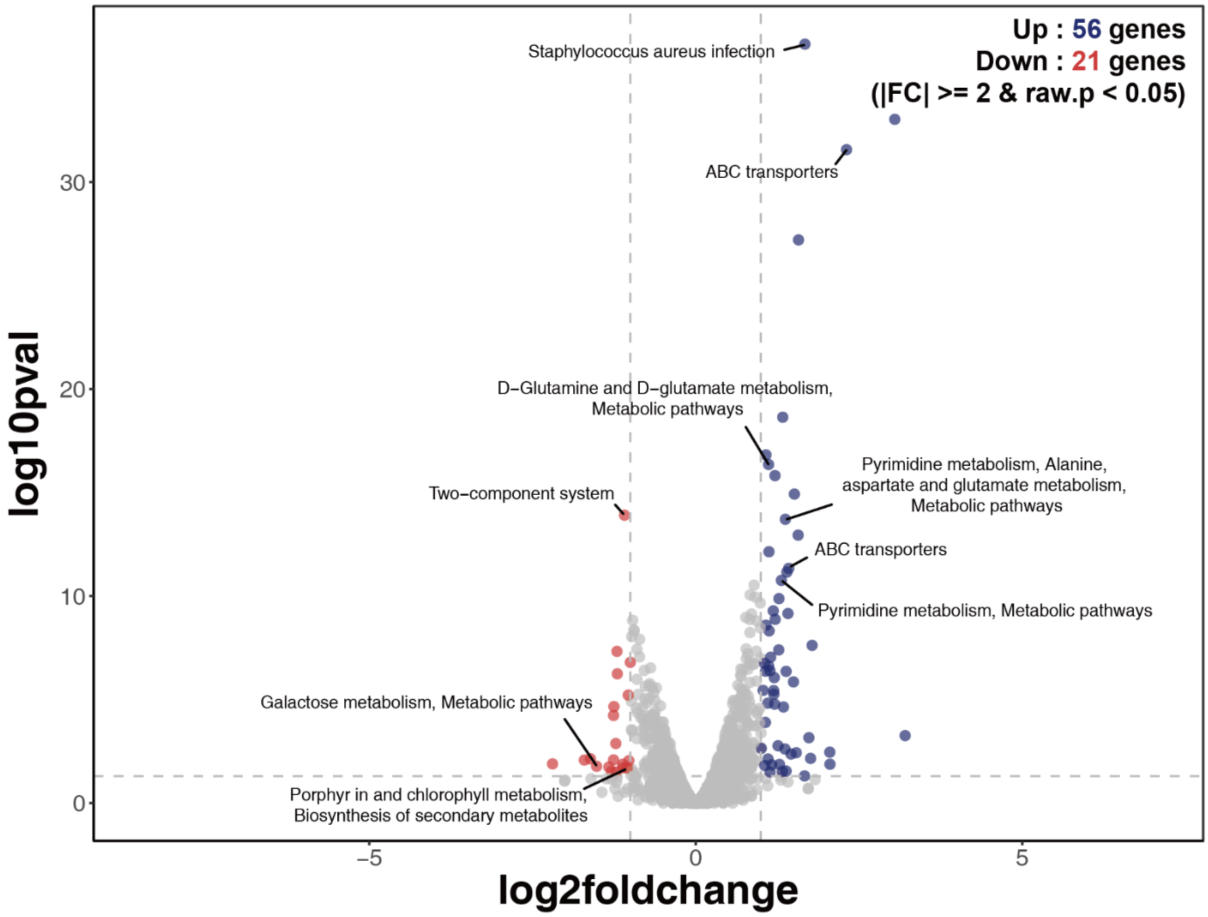

Fig. S5. *S. aureus* volcano plot of the log10 of the P value versus the log ratio fold changes in co-culturing with *M. restricta* versus axenic culturing. Thresholds are shown as dot lines indicating fold changes ≥ 2 and statistical significance defined by an adjusted p-value < 0.05. Functions described in the main text are labelled.

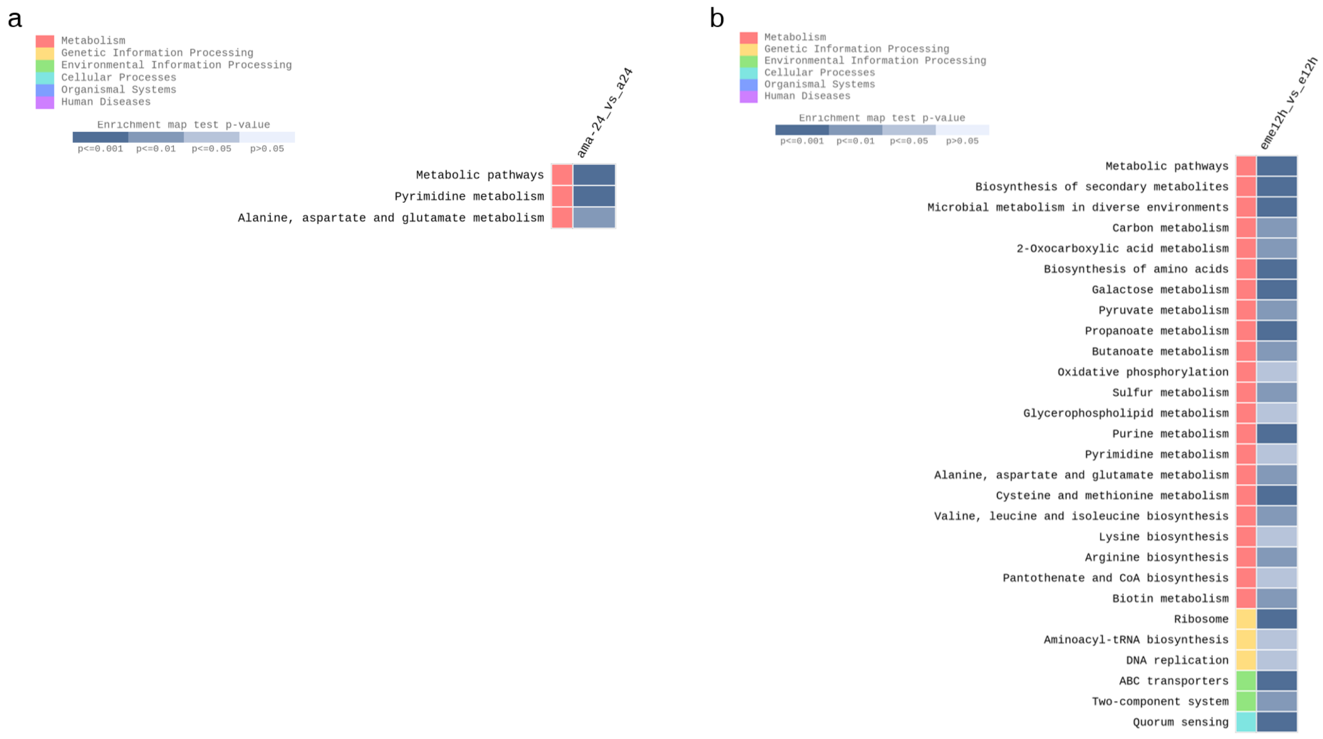

Fig. S6. Heat maps of KEGG pathway enrichment map analysis for (**a**) *Staphylococcus aureus* and (**b**) *Staphylococcus epidermidis*. The genes that were upregulated and downregulated in co-culturing, with $p < 0.05$ by the modified fisher's exact test, were subjected to KEGG database analysis. These were related to various biological pathways of metabolism (red), genetic information processing (yellow), environmental information processing (green), cellular processes (blue). The colors in the enrichment map represent the significant *p*-values of the geneset enrichment, with high significant values ($p \leq 0.001$) being colored in blue shade.

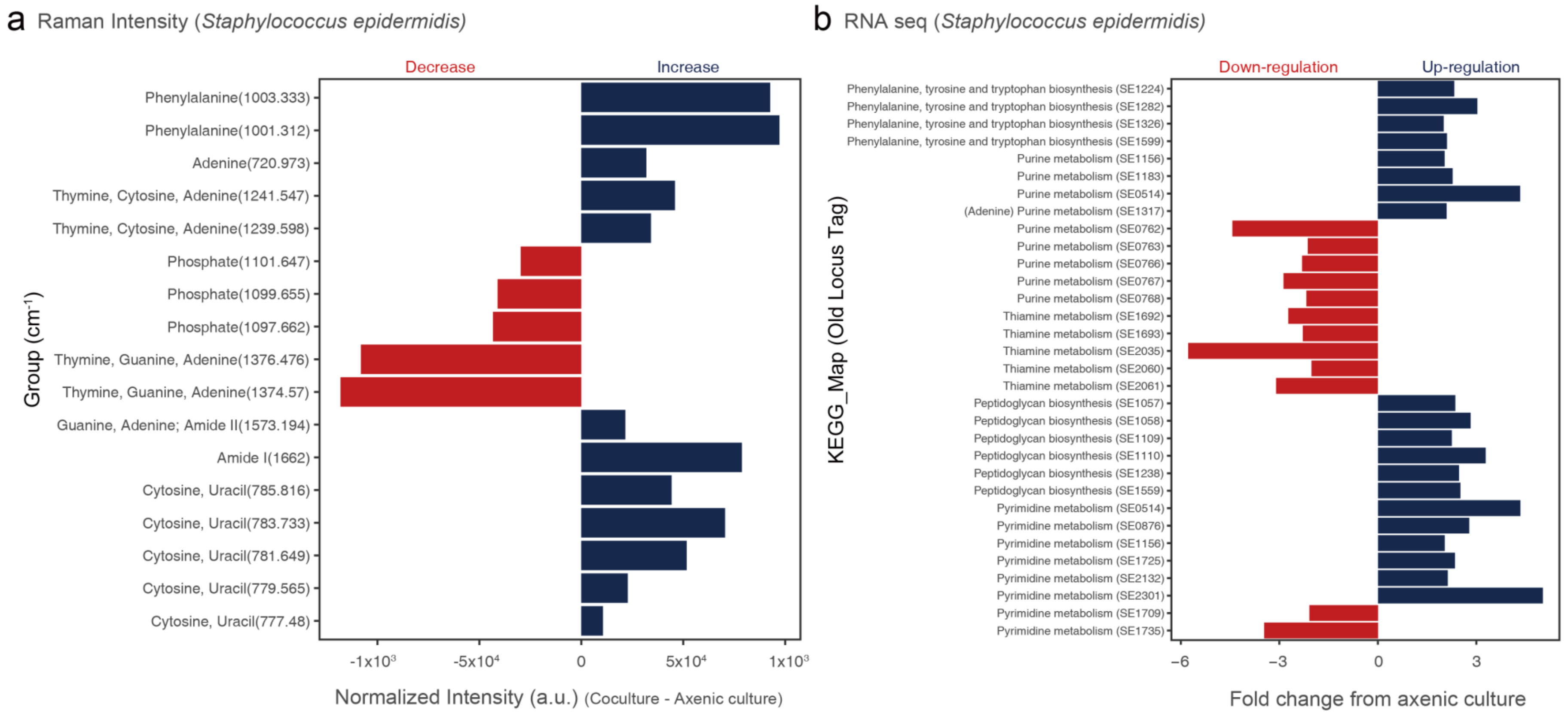

Fig. S7. Phenotype and genotype changes of *S. epidermidis* after co-culturing are matched. **a** The bars represent an increase or decrease in Raman intensity that has changed since co-culturing. **b** The bars represent the regulation of the same functional gene as the **(a)** Raman group after co-culturing. Increase is indicated in blue, and decrease is indicated in red.

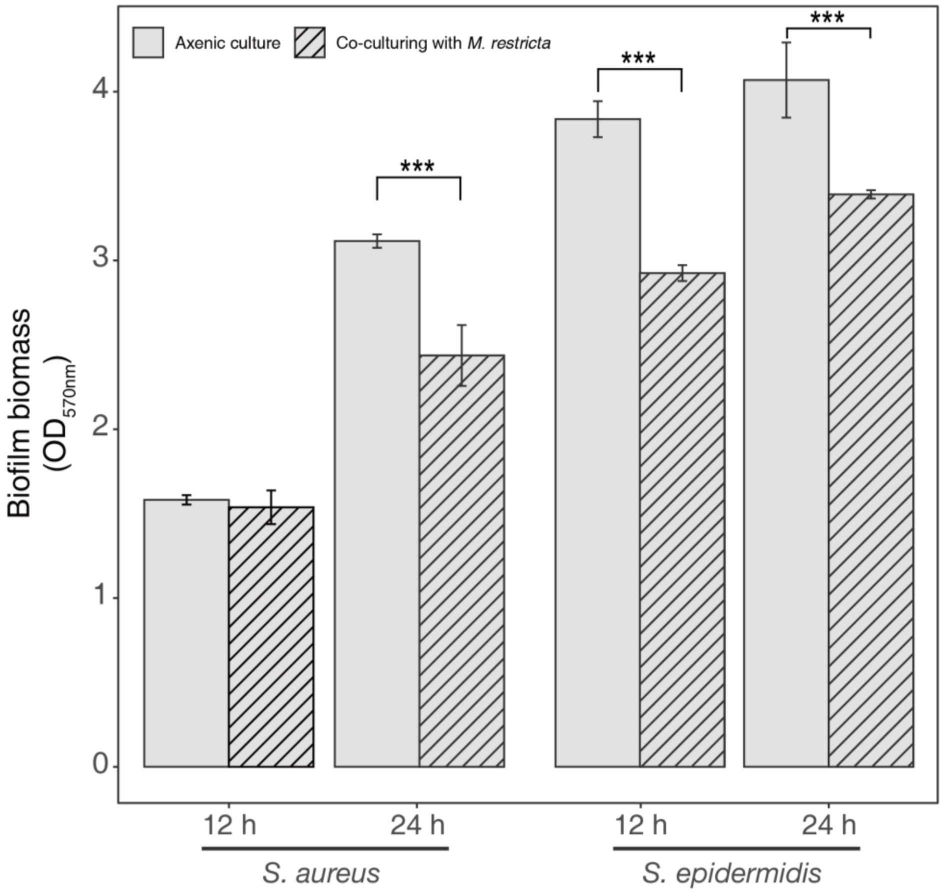

Fig. S8. Bacterial biofilm formation was visualized by crystal violet staining. Co-culturing with *Malassezia restricta* showed an increase in biofilm production. The error bars represent standard deviation of biofilm biomass. ***P < 0.001 Wilcoxon test (Axenic vs. Co-culture).

Supplementary Table 1. Assignment of the supplementary Raman bands. Raman peaks of bacterial spectra and attempt to assign bands based on the literature regarding vibrational energy bands.

Raman wavenumber (cm $^{-1}$)	Assignment	Group	Ref.	Fig. No.
723	Adenine		[60]	Fig S5(B)
749	pyrrole breathing mode	Cyt c.	[61]	Fig S4, Fig S5(A)
782	O-P-O breathing, Cytosine, Uracil	Cytosine, Uracil	[55]	Fig S2
783	Nucleic acids(C,T)		[60]	Fig S5(B)
838	DNA	DNA	[62]	Fig S4
853	ν(C–C) proline, ring breath. Tyr	Protein (glycogen, collagen)	[52, 53]	Fig S2, Fig S5(A)
897	COC str		[54]	Fig S4, Fig S5(B)
936	C–O–C linkage, C–C stretch., α-helix	Carbohydrate, protein	[53]	Fig S5(B)
972	CH_2 rock., C–C stretch., α-helix	Protein, lipid	[63]	Fig S4
1002	Phenylalanine	Phenylalanine, b-carotene	[55]	Fig S5(A), Fig S5(B)
1030-1130	Carbohydrates, mainly -C-C-(skeletal), C-O, def(C-O-H)		[56]	Fig S5(A)
1044	C-C, C-O, C-N str, C-O-H		[55]	Fig S5(B)
1079	PO2 str., (C–C) stretch., C–O	Nucleic acid, lipid, carbohydrates	[52]	Fig S4
1094	DNA: OPO-		[60]	Fig S5(B)
1098-1102	Phosphate, CC skeletal and COC str	Phosphate	[54]	Fig S5(A)
1123	CH Phe	Cytochrome	[52, 59]	Fig S5(B)
1127	=C-C= (unsaturated fatty acids in lipids)	lipids	[54]	Fig S4
1170	C–H in-plane bend. mode (Tyr), (CH) Phe	Protein	[59]	Fig S4
1209	C–C_6H_5 stretch., Phe, Trp	Protein	[52]	Fig S5(B)
1244	Amide III		[60]	Fig S5(B)
1246	Thymine, cytosine, adenine, ring	Thymine, cytosine, adenine	[54]	Fig S5(B)
1267	Lipids	Lipids	[64]	Fig S4, Fig S5(A)

1295	CH2 def		[51]	Fig S4
1295-1298	CH2 def, CH2 twist	Saturated lipid	[51]	Fig S5(A)
1304	Adenine, amide III	Adenine, amide III	[58]	Fig S2
1328	CH def		[55]	Fig S5(A)
1333	CH3CH2 def. of collagen	Nucleic acid, protein	[59]	Fig S5(B)
1375	Thymine, adenine, guanine	Thymine, adenine, guanine	[58]	Fig S2, Fig S5(A), Fig S5(B)
1441	Lipids	Lipids	[64]	Fig S4, Fig S5(A)
1482-1487	Nucleic acids	Nucleic acids	[56]	Fig S5(B)
1553	Tryptophane	Tryptophane	[57]	Fig S4
1573	Guanine, Adenine; Amide II, C=C, N–H def, and C–N str (amide II)	Guanine, Adenine; Amide II	[55]	Fig S2, Fig S5(A), Fig S5(B)
1582	Protein	Protein	[51]	Fig S4
1593	Protein	Protein	[51]	Fig S5(A)
1650-1680	Amide I	Amide I	[51]	Fig S5(B)
1655	Amide I	Amide I	[60]	Fig S2, Fig S4, Fig S5(A)
1662	Amide I	Amide I	[54]	Fig S5(A)
1663	Amide I	Amide I	[51]	Fig S2
